# Monte-Carlo simulations of external dose contributions from the surrounding ground areas of residential homes in a typical Northern European suburban area after a radioactive fallout scenario

**DOI:** 10.1038/s41598-020-71446-4

**Published:** 2020-09-08

**Authors:** Yvonne Hinrichsen, Robert Finck, Johan Martinsson, Christopher Rääf

**Affiliations:** Department of Translational Medicine, Medical Radiation Physics, Lund University, 205 02 Malmö, Sweden

**Keywords:** Mathematics and computing, Physics

## Abstract

The emissions of $^{137}\hbox {Cs}$ into the environment from the nuclear accidents in Chernobyl in 1986 and Fukushima in 2011 led to the need to decontaminate large areas to avert radiation doses to the population in the affected areas. To be able to perform cost-effective and sustainable remediation, knowledge is needed about how radiation doses can be minimized through optimized interventions such that the greatest possible reduction in radiation dose is obtained with the smallest possible negative impact on the area. Theoretical calculations have been performed to determine how radiation doses in single family houses in a typical Swedish residential suburb arise from a hypothetical $^{137}\hbox {Cs}$ deposition on the ground. The intention was to highlight how remediation of different parts of the surroundings affects the radiation dose to the residents in a particular property. A Monte Carlo model of the houses and the environment in a suburban area was set up to allow calculations of the dose contributions from different contaminated ground areas such as their own property, neighbouring properties, streets and surrounding recreational areas. Calculations were performed for eleven observation points inside different rooms of the house and one observation point in the garden outside the house, for four houses in the neighbourhood, and for two types of building construction material. The influence of the time spent in different rooms of the house and the contamination of areas surrounding the house was studied. The results show that in general the main dose contribution originates from their own property, but that a significant part (30–80%, depending on the observation point) can come from other areas, showing the importance of considering the surroundings in remediation actions. More detailed analysis of the results showed that the dose contribution from a source region is in general highly dependent on the position of windows in a brick house, whereas for a wooden house the distance to the source region is also of relevance.

## Introduction

The release of the radionuclide $^{137}\hbox {Cs}$ (half-life 30.05 years) into the environment from the nuclear power plant accidents in Chernobyl in Ukraine in 1986 and Fukushima in Japan in 2011 led to the need to decontaminate large inhabited areas to avert radiation doses to the population as it has been the most important radionuclide with regard to long-term effects of radioactive contamination in both accidents^1^. The cost of decontamination and the amount of waste that had to be disposed of were very large^2^. If similar accidents were to occur in the future, it would be important to implement remediation measures that are as efficient as possible, with as little negative impact as possible, for example, in terms of economic and social costs, as well as the amount of waste generated. According to the fundamental principles of radiation protection, measures must be both justified and optimized^3,4^. To achieve this, far-reaching knowledge is needed on how effective and costly different remediation measures are. Both measurements and calculations are required to investigate how radiation doses to individuals in the population are affected by various measures. A combination of measures should ultimately be chosen so as to give the greatest benefit in relation to the harm the measures entail. The calculations and results presented in this study are a step towards gaining the knowledge required for the optimization of remediation measures.

Based on experience gained following the two nuclear power plant accidents mentioned above, the contributions from external dose received by inhabitants in contaminated areas and the ingestion doses from the Chernobyl accident were estimated to be about equal in magnitude (based on 70 representative settlements in Belarus, Russia, and Ukraine), whereas the contribution of the external dose to the public affected by the Fukushima accident (based on data for the village of Kawauchi, Fukushima prefecture) was estimated to be of the order of 80–90% of the total dose^5^. Furthermore, measurements in the Bryansk region of the Russian Federation showed that inhabitants of multi-storey buildings received less than half the external dose received by inhabitants of single-storey houses in suburban areas^6^. Higher dose rates in suburban areas can be explained by the fact that grass and soil surfaces, which are much more common in suburbs where single family houses are found, retain $^{137}\hbox {Cs}$ more effectively than hard surfaces in urban environments, where weathering effects remove the deposited $^{137}\hbox {Cs}$ more rapidly, as described, for example, by one of the authors’ previous studies^7^. Based on these experiences, further studies of the radiation environment and resulting external doses to the inhabitants of urban and suburban areas are motivated to better understand the radiological consequences of hypothetical fallout events. This knowledge is fundamental for the choice of decontamination measures.

When devising the appropriate decontamination strategy, knowledge of the contributions to the external dose from different areas is important. For this purpose, we introduced the isodose concept as a method of describing the dose contributions at a specific observation point (inside or outside a building) from radioactive contamination of the surroundings such as different areas of the ground^8^. For example, the isodose line for 50 % surrounds the smallest area or areas that contribute 50% to the total external dose at a specific observation point. Furthermore, by considering a number of observation points inside the building, and assuming a specific residence time in each room, i.e. at each observation point, representative isodose lines can be determined for the residents taking into account the time they spend in the house. Isodose lines for high percentages of the dose contribution will most probably extend beyond the area of the inhabitants’ own property.

The dose contributions from radioactive contaminants deposited on various urban surfaces have been analysed in a case study by Andersson et al.^9^. The general findings were presented in terms of 20-year post-Chernobyl fallout dose contributions from various types of surfaces, based on a contamination of $1\,\hbox {MBq m}^{-2}$ of $^{137}\hbox {Cs}$ on a grassed reference surface, to people living in a dry contaminated area in the Bryansk region. It was shown that a large contribution, 16–22 mSv/($\hbox {MBq m}^{-2} {}^{137}\hbox {Cs}$), originated from the soil and grass areas, for example, the gardens of brick houses, and that value could be four times higher for wooden houses. Contaminated roofs can contribute significantly to the external dose to inhabitants, 7–10 mSv/($\hbox {MBq m}^{-2}{}^{137}\hbox {Cs}$), but only when the inhabitants lived on the top floor of a dwelling with a light roof construction. This contribution to the external dose is significantly smaller if the building has an attic and less than 1 mSv/($\hbox {MBq m}^{-2}{}^{137}\hbox {Cs}$) to persons not residing on the top floor. The contamination of walls is generally very low compared to the ground, and their contribution to the dose is about 0.3–1 mSv/($\hbox {MBq m}^{-2}{}^{137}\hbox {Cs}$) for brick walls and about 1–2 mSv/($\hbox {MBq m}^{-2}{}^{137}\hbox {Cs}$) for very thin wooden walls. The contribution from radioactive contamination of trees and shrubs can be vary considerably, depending on the shielding provided by the house, i.e. around 2–3 mSv/($\hbox {MBq m}^{-2}{}^{137}\hbox {Cs}$) for a house that is not very well shielded and less than 0.5 mSv/($\hbox {MBq m}^{-2}{}^{137}\hbox {Cs}$) for a well-shielded building. Many of these dose contributions are of the same order of magnitude. However, this would not have been the case if contamination had occurred during heavy rain, which would have left comparatively little contamination on other outdoor surfaces than to permeable horizontal surfaces (grass/soil), thus resulting in significantly lower values than in case of dry deposition.

To the best of our knowledge, previous studies have only addressed external dose contributions from unfractionated environmental surfaces (e.g. by Meckbach et al.^10,11^, Jacob and Meckbach^12^, Kis et al.^13,14^, Salinas et al.^15^, Dickson et al.^16^, Dickson and Hamby^17^, and Hinrichsen and Andersson^18^), such as the whole ground area. In contrast, we have applied the isodose concept to study the external dose contributions inside typical Northern European house models from contamination on the ground with a resolution of $1\,\hbox {m}^{2}$^19^.

The purpose of the present study was to extend the concept of isodose lines to model an entire neighbourhood of single-family houses in a typical Swedish environment, which can also be said to be representative of suburban areas in other Northern European countries. Typical single-storey wooden houses and brick houses were studied based on the house models described previously^19^. The contributions to the external dose in different rooms of the house, and in the garden outside the house, from $^{137}\hbox {Cs}$ deposition on the ground of their own property, adjacent properties, streets and remote areas were calculated to show how the dose contributions vary from room to room, and how decontamination of one’s own property and surrounding properties helps to reduce future doses.

## Results

**Figure 1:**
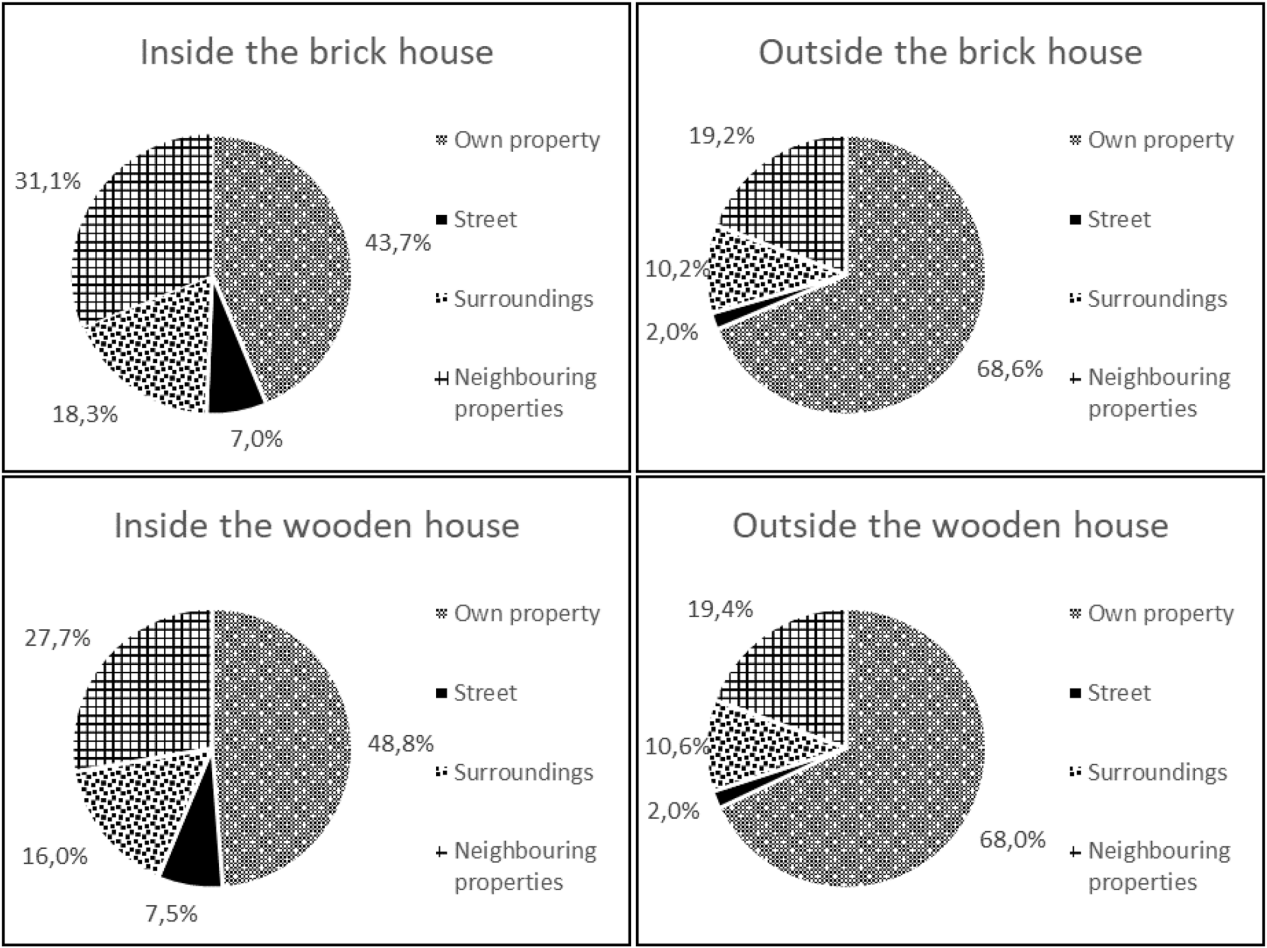
Average dose contributions from the various source areas to the observation points inside and outside the brick and the wooden houses.

**Figure 2:**
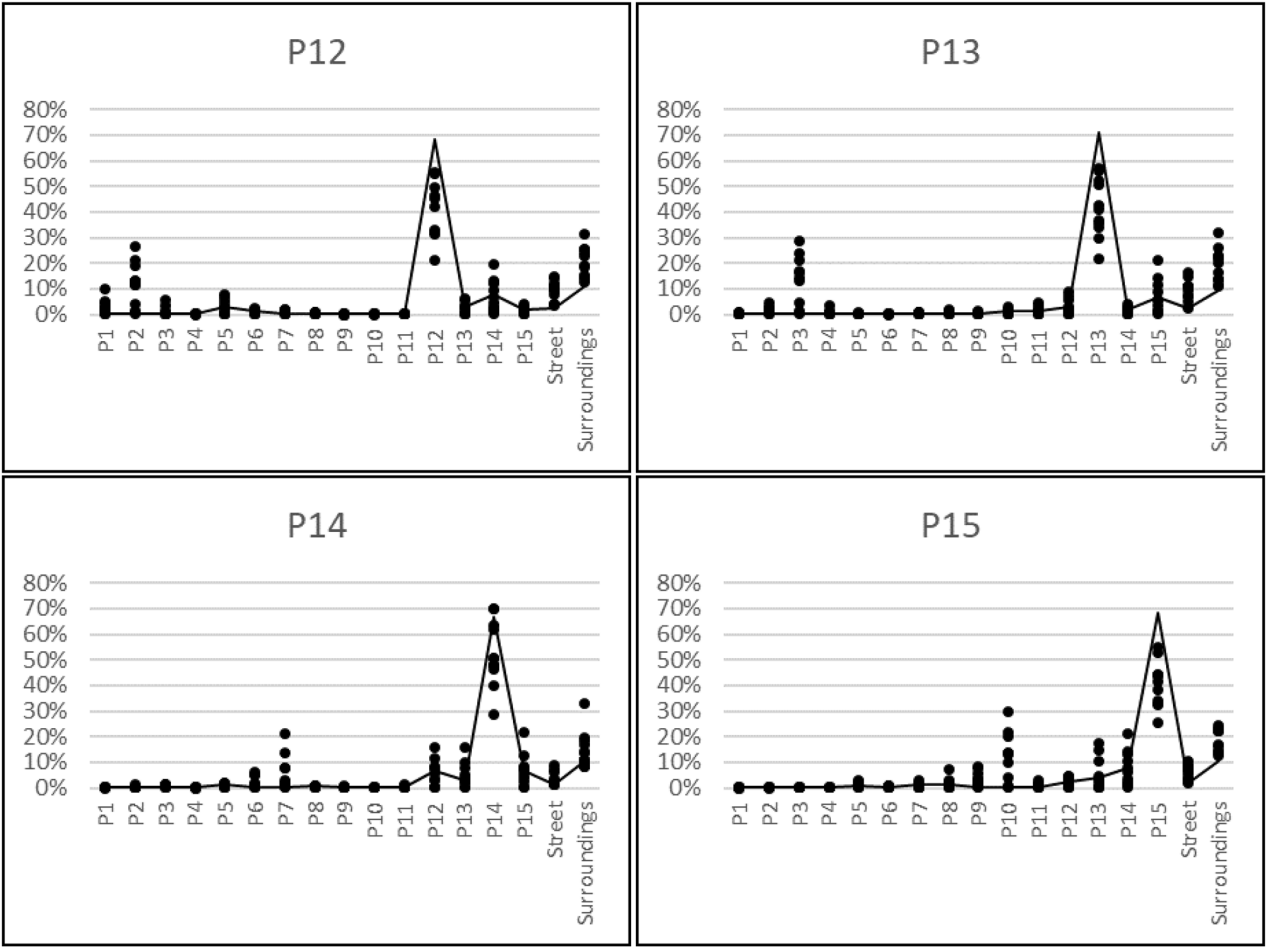
Dose contributions from the various source areas to observation points inside (dots) and outside (line) the brick house for properties P12–P15.

**Figure 3:**
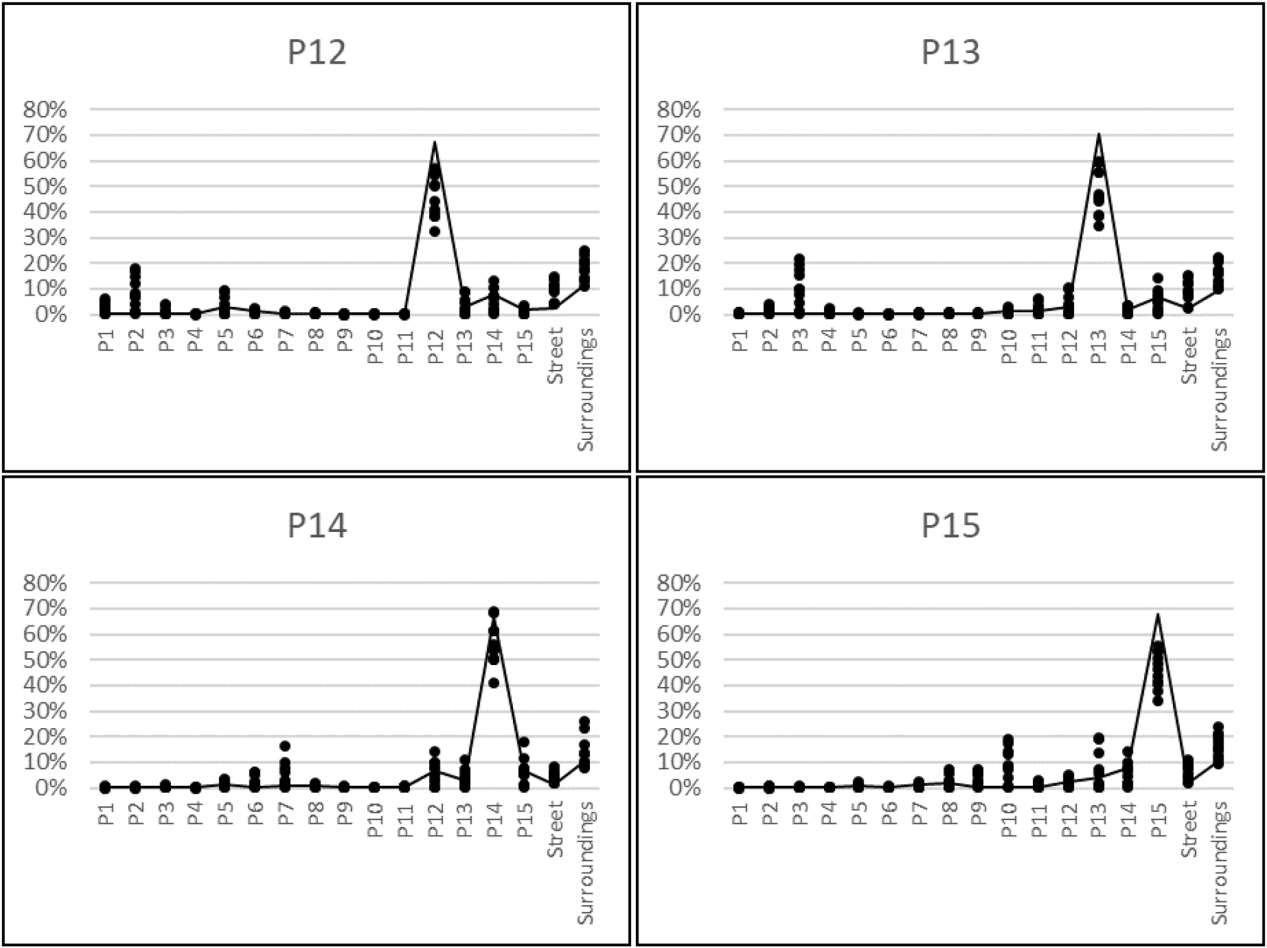
Dose contributions from the various source areas to observation points inside (dots) and outside (line) the wooden house for properties P12–P15.

The resulting average dose contributions from a typical Northern European neighboorhood as described in more detail in the methods section (see Fig. 6) are presented in Fig.  1 for properties P12-P15. More detailed data for the observation points inside the house and the value for the observation point outside the house are plotted in Fig.  2 for the model with brick houses and in Fig.  3 for the model with wooden house.

### Dose contributions from one’s own property

From the figures given above, it can be seen that the dose contribution from radioactive contamination of one’s own garden inside one house in the model with brick houses is on average just over 40% for all studied houses except P14, which is positioned more centrally in its own garden. The results are similar for the model with wooden houses, although the average value is slightly higher than for the model with brick houses, over 45%, as the wooden construction material provides less shielding, and the dose contribution is thus higher from areas that are closer to the observation points. The dose contributions from radioactive ground contamination on one’s own property at the observation points outside the houses are up to 70 % higher than at observation points inside the houses. This can be explained by the lower effect of geometry shielding (as defined the Office of civil defence^20^) for observation points outside the house, and thus the contaminated areas closer to the observation point have a greater effect on the dose rate^21^. This also explains why there are no significant differences between these fractions for the model with brick houses and wooden houses.

### Dose contributions from the street

The dose contribution from radioactive contamination of the street at observation points inside the house varies between 1.5% and 16.5%. This dose contribution is higher for properties P12 and P13 than for properties P14 and P15, as P12 and P13 have two outer walls close to the street, while P14 and P15 do not. No significant differences were found between the brick and the wooden houses in the simulated scenario. The dose contribution from the street to outdoor observation points fluctuates around 2% and, as for the indoor observation points, the values are higher for properties P12 and P13 than for properties P14 and P15, with no significant differences between the with brick and wooden houses. In general, the long-term dose contribution from radioactive contaminants on the street is of less concern as weathering processes on these surfaces have been found to be rapid (e.g. by Hinrichsen and Andersson^7^).

### Dose contributions from neighbouring properties

In the next step, the dose contributions from radioactive contaminants deposited on neighbouring properties were analysed. The average contributions from one neighbouring property to observation points inside the house vary between 0.06% and 12.87% in the brick house and between 0.06% and 9.86 % in the wooden house. At the outdoor observation points, the values vary between 0.06% and 7.92% for the model with brick houses and between 0.06% and 7.82 % for the model with wooden houses. The variation is due to the distance from the source region to the observation point and shielding by other houses in the line of sight between a source region and the observation point. In contrast to contamination on the ground of one’s own property, the dose contribution from neighbouring properties is higher for the model with brick houses than wooden houses. This is due to the relatively higher dose contribution from areas that are closer to the observation point in the wooden house, since its construction material provides less shielding than for brick houses. Thus, as the dose contribution from one’s own property is higher for the wooden house, the remaining fraction of the dose from other areas is lower. The same effect explains the values for the observation points outside the houses.

The ten highest values, in terms of the dose contribution to an observation point in a specific room from radioactive ground surface contamination of neighbouring properties, are listed in Table 1 for the model with brick houses and wooden houses. From the results in this table it is apparent that for the model with brick houses the main factor governing the dose contribution is whether the observation point is in a room with a window in direction facing the respective source region. This is also the case for the wooden house, but the distance between the source and observation point is of greater importance than for the brick house, again because the wooden construction material provides less shielding against radiation than the brick construction material. This also explains why the dose contributions listed in the table are generally higher for the model with brick houses than for the wooden houses.

**Table 1:** List of the ten highest dose contributions to an observation point in a specific room of a property from radioactive contamination of the ground of another property, for the model with brick houses and the wooden houses.

Source region	Indoor observation point		Dose contribution	
	Property	Room	Brick house (%)	Wooden house (%)
P2	P12	OP4	26.67	18.23
P2	P12	OP6	21.43	16.96
P3	P13	OP4	28.79	21.63
P3	P13	OP6	23.72	19.62
P3	P13	OP8	21.35	17.18
P15	P14	OP4	21.96	18.16
P7	P14	OP8	21.25	16.61
P13	P15	OP1	14.98	19.10
P14	P15	OP2	21.41	14.34
P13	P15	OP3	17.62	19.58
P10	P15	OP4	29.86	19.25
P10	P15	OP6	21.79	17.62

### Dose contributions from the surrounding areas

The dose contributions from radioactive contamination of surrounding areas of this suburban neighbourhood to the observation points inside the houses vary between 8.4% and 32.8% for the model with brick houses and between 8.0% and 26.0% for the model with wooden houses. The values at the observation points outside the houses vary between 9.4% and 11.8%. The dose contribution from the surrounding areas is mostly the result of the large size of the source area, and can be regarded as background radiation exposure, and therefore difficult to assign to specific source areas.

### Dose contributions according to typical occupancy of a resident

**Figure 4:**
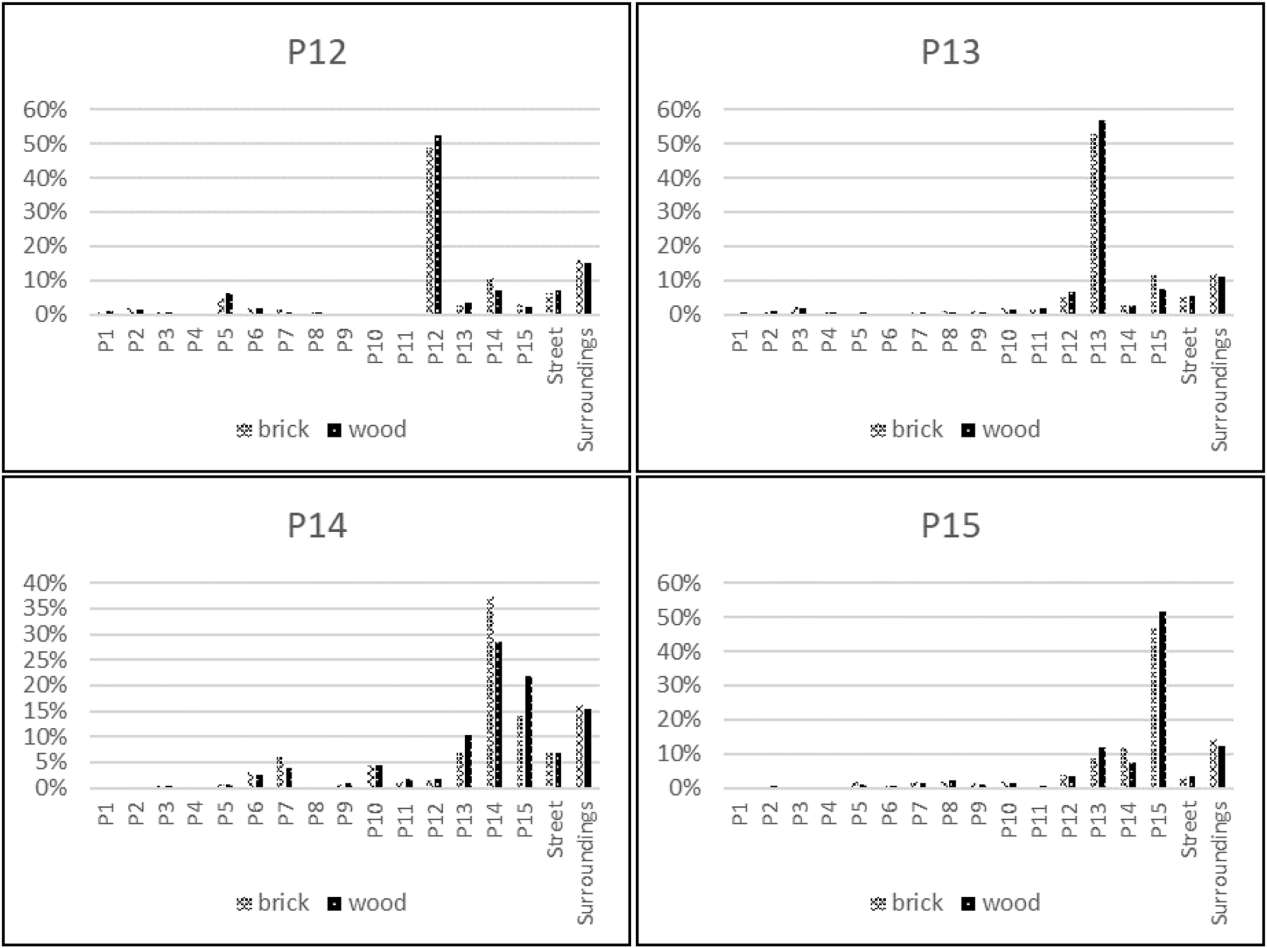
Dose contributions from the various source areas inside the brick and wooden houses on properties P12–P15 for typical resident occupancy.

To determine the dose contribution from each source region for the entire house, occupancy data for a typical resident were used. The dose contribution to the observation point in one room was multiplied by the fraction of time spent in that room relative to the amount of time spent in the entire house, over a 24-h period. The results are presented in Fig. 4 for both the model with brick house and wooden houses. The most remarkable result was observed for property P14 in the wooden house scenario, where the dose contribution from radioactive ground surface contamination of the neighbouring property P15 was almost 22%. This high value can be explained by the position of the bedroom (OP1), as it faces property P15, and the fact that the highest fraction of time spent in the house is in the bedroom.

Although this is only one example, this configuration of houses is realistic for many Northern European suburbs, and this kind of scenario is thus likely in a real fallout situation. It also serves to illustrate that the potential dose reduction by decontamination of a single property may still be dependent on how well such procedures are carried out on neighbouring properties.

## Discussion

The results presented in this study, illustrate how radioactively contaminated ground areas in a typical Northern European suburban neighbourhood contribute to the external dose inside and outside of typical Northern European dwellings. In general, the main dose contribution originates from one’s own property, but a significant part (30–80%, depending on the observation point) can come from other areas. The dose contribution from one’s own property is higher inside wooden houses than inside brick houses, and is generally highest outside the house. The external dose contribution to a specific room inside the house is highly dependent on the position of the windows in a brick house, whereas for a wooden house the distance to the source region is also of importance, as this building material provides less shielding. These principles can be also applied to other positions inside the house and have been already found in one of our previous studies^19^, in which dose contributions have been studied in higher resolution for a smaller area. Furthermore, this study shows the importance of considering the occupancy of the various rooms in the house (e.g. the bedroom), as the time spent in a room strongly influences the external dose contribution from the surrounding area outside the house.

It should be borne in mind that this study represents an extreme case in which gamma-emitting contamination on the ground surface was modelled. In reality, radioactive contamination penetrates into the ground, and will migrate downwards over time^7,22^. According to previous findings (e.g. by Hinrichsen et al.^19^), the contaminated areas that contribute most to the dose at a certain position move closer to the observation point, as the ground contamination migrates into the ground as the contributions from more remote areas become less significant due to the attenuation of the gamma flux by the ground in the line of sight between the source area and the observation point. Therefore, the external dose contribution from the neighbouring surfaces in this study can be assumed to be an upper limit and, in reality, the dose contribution from contaminated areas of one’s own property may be higher. Moreover, in reality the contamination would not be homogeneous as assumed in this study, but the impact of variability in contamination with its dependence on building material in regard to dose contribution of certain contaminated areas has already been studied and discussed in one of our previous publications^19^.

In conclusion, knowledge about the various factors that influence the dose contribution from contaminated areas in a suburban neighbourhood is important when assessing the local radiation environment in affected residential areas, and increases the decision base for the application of countermeasures.

## Methods

**Figure 5:**
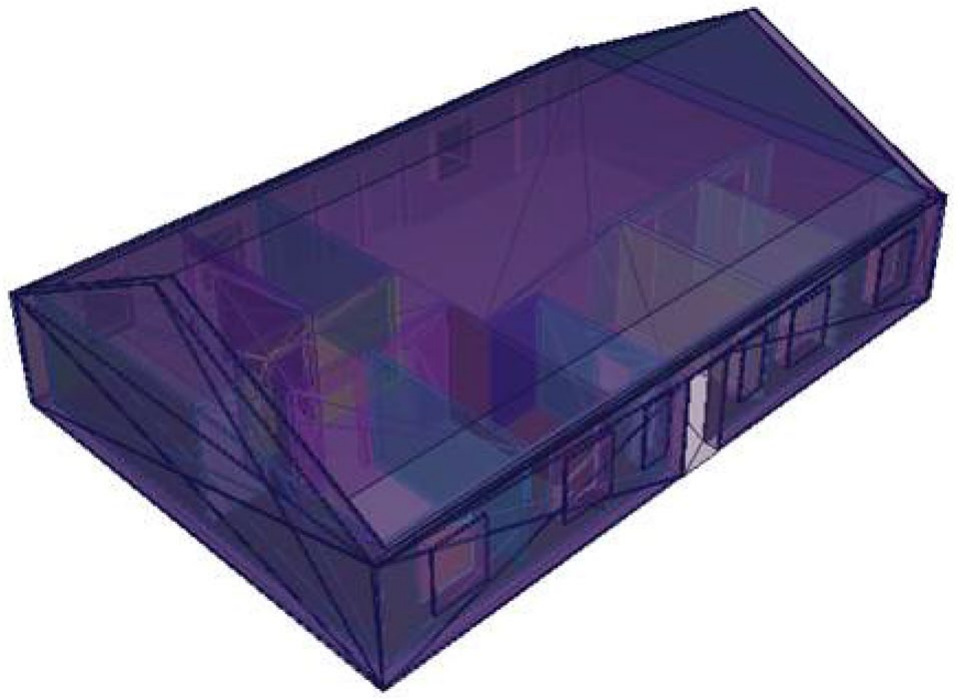
Birds-eye view of a typical single-storey Swedish house of brick or wooden construction material^19^.

The Monte Carlo calculations for a typical Northern European suburban area were performed with the transport code MCNP6.2^23^, using the nuclear cross-section data set ENDF/B-VII.0^24^. Among other processes, the code accounts for photon creation and loss through relevant mechanisms such as bremsstrahlung, fluorescence, Compton scattering, photon capture, pair production and p-annihilation. The complex 3-dimensional models of typical Northern European one-storey houses as described previously^19^, were defined through a combinatorial geometric technique, based on construction drawings and descriptions of an actual wooden and a brick house made available by the Urban Planning Department of the Municipality of Hässleholm in Sweden (Stadsbyggnadskontoret, Hässleholms kommun) (Fig. 5). Further details, such as the elemental composition of buildings materials can be found in our previous publication^19^.

**Figure 6:**
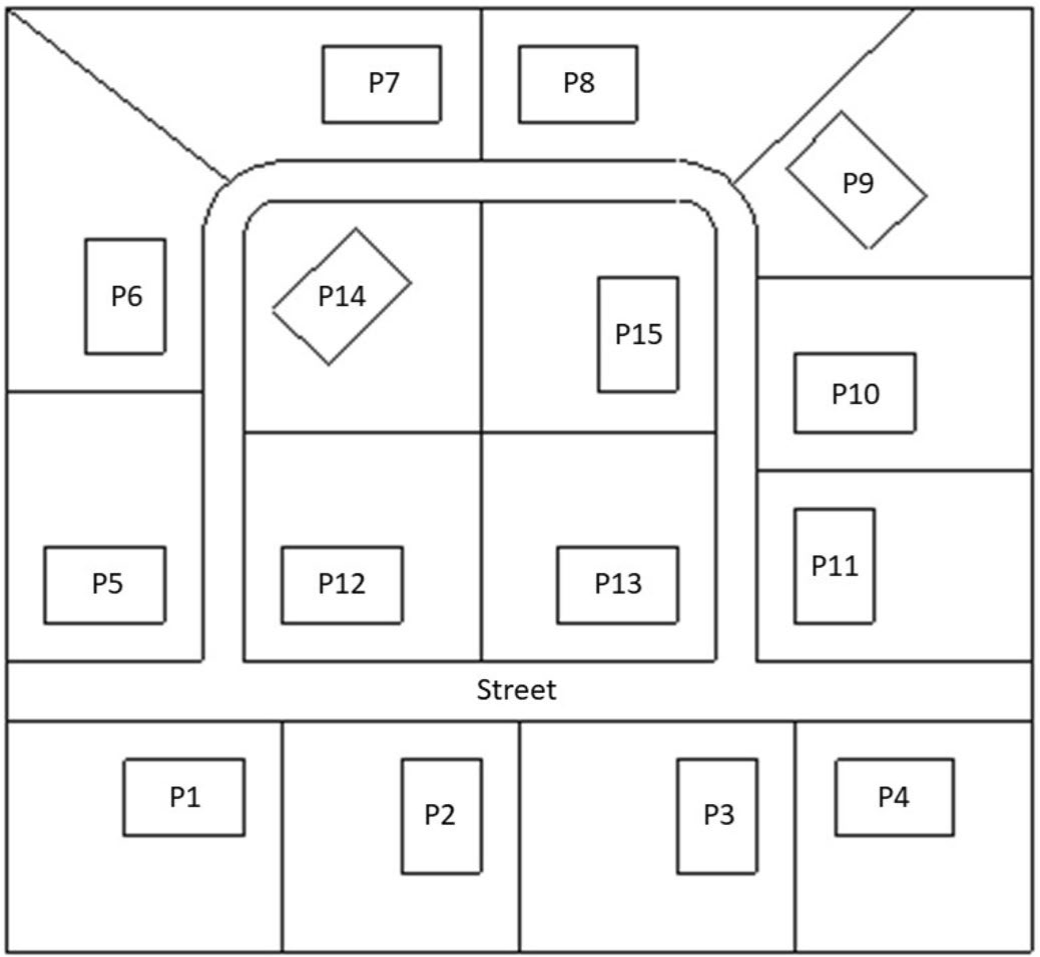
Overview of a typical Swedish suburban neighbourhood consisting of 15 properties (P1–P15), showing their respective garden boundaries and the street.

The models for the brick house and the wooden house were multiplied and set up to mimic a typical Northern European suburban area including 15 properties (one model including only brick houses and one model including only wooden houses) and a connecting street, covering an area of $16\,250\,\hbox {m}^{2}$ (Fig.  6). The size of the properties varied between $875\,\hbox {m}^{2}$ and $1050\,\hbox {m}^{2}$ and each house covered an area of $150\,\hbox {m}^{2}$. The street was modelled as an 18 cm thick layer of asphalt with a density of $2.5784\,\hbox {g/cm}^{3}$ and a material composition of 13.40% H, 11.01% C, 0.05% N, 49.82% O, 0.88% Na, 1.55% Mg, 3.28% Al, 14.31% Si, 0.15% S, 0.76% K, 3.66% Ca, 0.12% Ti, 0.01% Mn, 0.98% Fe, and 0.01% Pb according to material compendium (Material 19^25^). This artificial suburban area was based on satellite images of residential areas in Hässleholm, Sweden in 2019.

A radioactive gamma-emitting source with an energy of 0.662 MeV was used in the calculations as this is the energy of the gamma-rays emitted by a metastable isomer of $^{137}\hbox {Ba}$ as product of 94.4% of the beta minus decays of the fission product $^{137}\hbox {Cs}$, which is the radionuclide of greatest concern in connection with the Chernobyl and Fukushima nuclear power plant accidents (e.g. see Imanaka et al.^1^). The gamma-emitting source regions were defined at ground surface level, as it was found in our previous study that a source at this level represents an upper extreme in terms of dose contributions from remote areas at the observation point, compared to lower source levels assuming ground penetration^19^. As deposition migrates deeper into the soil, the soil shields the gamma radiation. This effect is of greater importance for areas at greater distances from the observation point, as there is more soil in the line of sight between the source area and the observation point, thus the isodose lines are closer to the observation point at deeper soil levels compared to a source area at ground surface level. In reality, contaminated areas closer to the observation point would have a higher contribution and contaminated areas further away from the observation point a lower contribution, due to the roughness of the ground surface. The shapes of the source areas were defined according to the gardens (excluding the area covered by the houses) and the street (Fig. 6), and an infinite area surrounding the defined area.

**Figure 7:**
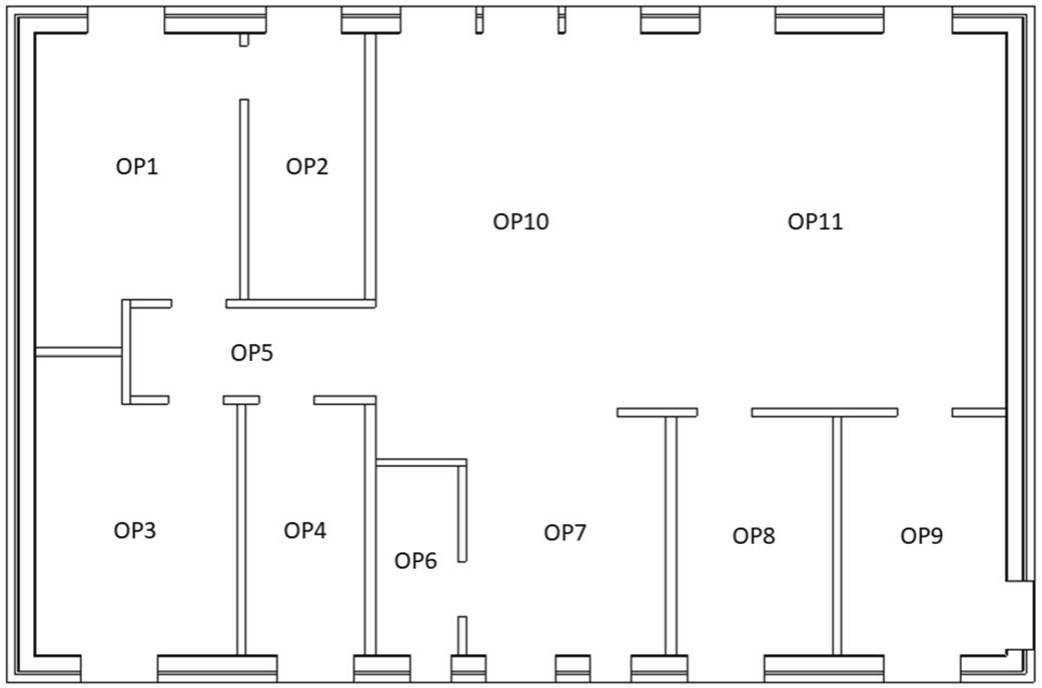
Observation points inside a typical Swedish house^19^.

The detector regions were defined as air-filled spheres with a diameter of 30 cm, positioned 1 m above ground level on properties P12-P15 and located at the observation points (OP) in our previous study^19^ in the middle of the different rooms inside the house (Fig. 7) added by an observation point in the middle of the garden of the house P12-P15 (Fig. 6). The numbers and energies of the gamma ‘particles’ passing through these detector regions were determined with the Monte Carlo code. The gamma fluence was transformed into air kerma free-in-air using conversion coefficients^26^. All calculations were performed for $4\cdot 10^7$ source particles without further variation reduction techniques. The statistical quality of all results were assured by ten statistical tests that are provided by the code and performed for each detector region.

To determine the dose contribution from each source region to a specific observation point, the air kerma value for combinations of source region and observation point was simply divided by the sum of the air kerma values from all source regions to this particular observation point. Thus, all the dose contributions determined in this study are based on air kerma values.

In order to determine the dose contribution from each source region to the entire house, the occupancy of a typical resident must be considered. Occupancy factors were chosen based on the data published in the European EXPOLIS project^27–29^, in which thousands of people in seven European cities (Athens, Basel, Grenoble, Helsinki, Oxford, and Prague), were studied with respect to their time budgets, and the hours they spent in various microenvironments, as described previously^19^. From these data it was found that people spend about 14 h indoors at home. About 1 h is spent in the kitchen preparing food (Room OP9 in Fig.  7), about 1 h eating^30^ (i.e. in the dining room, Room OP11), about 8 h sleeping^31^ (i.e. in the bedroom, Room OP1), and about 0.5 h in the bathroom^32^ (Room OP2), leaving about 3.5 h which it is assumed is spent in the living room (Room OP10). The remaining 10 h of the day have not been taken into account as they may be spend at other places as e.g. at work.

## Data Availability

The datasets generated during and analyzed during the current study are available from the corresponding author on reasonable request.

## References

[1] Imanaka T, Hayashi G, Endo S (2015). Comparison of the accident process, radioactivity release and ground contamination between Chernobyl and Fukushima-1. J. Radiat. Res..

[2] Munro A (2013). The economics of nuclear decontamination: assessing policy options for the management of land around Fukushima dai-ichi. Environ. Sci. Policy.

[3] ICRP. *The 2007 Recommendations of the International Commission on Radiological Protection*. Annals of the ICRP, ICRP Publication 103 (2007). 10.1016/j.icrp.2007.10.003 18082557

[4] ICRP. *Application of the Commission’s Recommendations to the Protection of People Living in Long-term Contaminated Areas after a Nuclear Accident or a Radiation Emergency*. Annals of the ICRP, ICRP Publication 111 (2009). 10.1016/j.icrp.2009.09.008 20472181

[5] Howard BJ, Fesenko S, Balonov MI, Pröhl G, Nakayama S (2017). A comparison of remediation after the Chernobyl and Fukushima Daiichi accidents. Radiat. Prot. Dosimetry..

[6] IAEA. *Present and Future Environmental Impact of the Chernobyl Accident*. IAEA-TECDOC-1240 (2001).

[7] Hinrichsen Y, Andersson KG (2019). European decision support modelling of long-term external doses received in inhabited areas contaminated by a nuclear power plant accident - 2: post deposition contaminant mobility on outdoor surfaces. J. Environ. Radioact..

[8] Hinrichsen Y, Finck R, Rääf C, Andersson KG (2018). Introducing the concept of the isodose for optimisation of decontamination activities in a radioactive fallout scenario. J. Radiol. Prot..

[9] Andersson, K. G., Jones, J. A. & Charnock, T. W. Chapter 6: Estimation of doses in inhabited areas. In *Airborne Radioactive Contamination in Inhabited Areas, Vol. 15 of Radioactivity in the Environment* (ed. Andersson, K. G. ) 147–185 (Elsevier, 2009).

[10] Meckbach R, Jacob P, Paretzke HG (1987). Shielding of gamma radiation by typical European houses. Nuclear Instrum. Methods Phys. Res. A.

[11] Meckbach R, Jacob P, Paretzke HG (1988). Gamma exposures due to radionuclides deposited in urban environments. Part I: Kerma rates from contaminated urban surfaces. Radiat. Protect. Dosimetry.

[12] Jacob P, Meckbach R (1987). Shielding factors and external dose evaluation. Radiat. Prot. Dosimetry..

[13] Kis, Z., Eged, K., Voigt, G., Meckbach, R. & Müller, H. Guidelines for planning interventions against external exposure in industrial area after a nuclear accident. Part II: Calculation of doses using Monte Carlo method. GSF-Bericht 02/03 (2003).

[14] Kis Z, Eged K, Voigt G, Meckbach R, Müller H (2004). Modeling of an industrial environment: external dose calculations based on Monte Carlo simulations of photon transport. Health Phys..

[15] Salinas ICP, Conti CC, Rochedo ERR, Lopes RT (2006). Gamma shielding factor for typical houses in Brazil. Radiat. Prot. Dosimetry..

[16] Dickson ED, Hamby DM, Eckerman KF (2015). Contaminant deposition building shielding factors for US residential structures. J. Radiol. Prot..

[17] Dickson ED, Hamby DM (2016). Building protection- and building shielding-factors for environmental exposure to radionuclides and monoenergetic photon emissions. J. Radiol. Prot..

[18] Hinrichsen Y, Andersson KG (2019). Kerma conversion factors for modern glass buildings in radioactively contaminated areas. J. Radiol. Prot..

[19] Hinrichsen Y, Finck R, Martinsson J, Rääf C, Andersson KG (2019). Influence of the migration of radioactive contaminants in soil, resident occupancy, and variability in contamination on isodose lines for typical Northern European houses. Sci. Rep..

[20] OCD. Office of civil defence, Shelter design and analysis—Volume 1 fallout protection (1962).

[21] Hinrichsen, Y. Improving the decision base for nuclear and radiological emergency management by modeling external radiation exposure. PhD thesis (2019).

[22] Kirchner G, Strebl F, Bossew P, Ehlken S, Gerzabek MH (2009). Vertical migration of radionuclides in undisturbed grassland soils. J. Environ. Radioact..

[23] Werner, C. J., Bull, Jeffrey S., Solomon, C. J., Brown, Forrest B., McKinney, G. W., Rising, M. E., Dixon, D. A., Martz, R. L., Hughes, H. G., Cox, L. J., Zukaitis, A. J., Armstrong, J. C., Forster, R. A. & Casswell, L. MCNP Version 6.2 Release Notes (2018).

[24] Chadwick, M., Obložinský, P., Herman, M., Greene, N., McKnight, R., Smith, D., Young, P., MacFarlane, R., Hale, G., Frankle, S., Kahler, A., Kawano, T., Little, R., Madland, D., Moller, P., Mosteller, R., Page, P., Talou, P., Trellue, H., White, M., Wilson, W., Arcilla, R., Dunford, C., Mughabghab, S., Pritychenko, B., Rochman, D., Sonzogni, A., Lubitz, C., Trumbull, T., Weinman, J., Brown, D., Cullen, D., Heinrichs, D., McNabb, D., Derrien, H., Dunn, M., Larson, N., Leal, L., Carlson, A., Block, R., Briggs, J., Cheng, E., Huria, H., Zerkle, M., Kozier, K., Courcelle, A., Pronyaev, V. & van der Marck, S., 2006. ENDF/B-VII.0: Next generation evaluated nuclear data library for nuclear science and technology. *Nuclear Data Sheets.* **107** (12), 2931 – 3060 (2006), evaluated Nuclear Data File ENDF/B-VII.0.

[25] McConn Jr, R., Gesh, C., Pagh, R., Rucker, R., Williams III, R. Radiation portal monitor project—Compendium of material composition data for radiation transport modeling. Pacific northwest national laboratory (2011).

[26] ICRP. Conversion coefficients for radiological protection quantities for external radiation exposures. *Annals of the ICRP*. **40**(2), 1 – 257, ICRP Publication 116 (2010). 10.1016/j.icrp.2011.10.001 22386603

[27] Jantunen MJ, Hanninen O, Katsouyanni K, Knoppel H, Kuenzli N, Lebret E, Maroni M, Saarela K, Sram R, Zmirou D (1998). Air pollution exposure in European cities: the EXPOLIS study. J. Expo. Anal. Environ. Epidemiol..

[28] Rotko T, Oglesby L, Kunzli N, Jantunen MJ (2000). Population sampling in European air pollution exposure study, EXPOLIS: comparisons between the cities and representativeness of the samples. J. Expo. Anal. Environ. Epidemiol..

[29] Schweizer, C. EXPOLIS annex (final report of WP1 of the EXPOLIS study funded by CEFIC) (2004)

[30] USDA. United States Department of Agriculture, How much time do Americans spend eating? (2018). https://www.thefreelibrary.com/How+much+time+do+Americans+spend+eating%3f-a0190462486/.

[31] OECD. OECD database, The Organisation for Economic Co-operation and Development (OECD) (2018). http://www.oecd.org/els/family/database.htm.

[32] The Scotsman. How long do we spend in bathroom? Published Friday 4 January 2008 (2008). https://www.scotsman.com/news/how-long-do-we-spend-in-bathroom-1-189-years-1-1072528.

